# Supplementary material for: Structural comparison of homologous protein-RNA interfaces reveals widespread overall conservation contrasted with versatility in polar contacts
Source: PLoS Comput Biol. 2024 Dec 3;20(12):e1012650. doi: 10.1371/journal.pcbi.1012650 (PMC11642956; doi:10.1371/journal.pcbi.1012650)
Supplement: S1 Fig — (PDF) [file pcbi.1012650.s001.pdf]

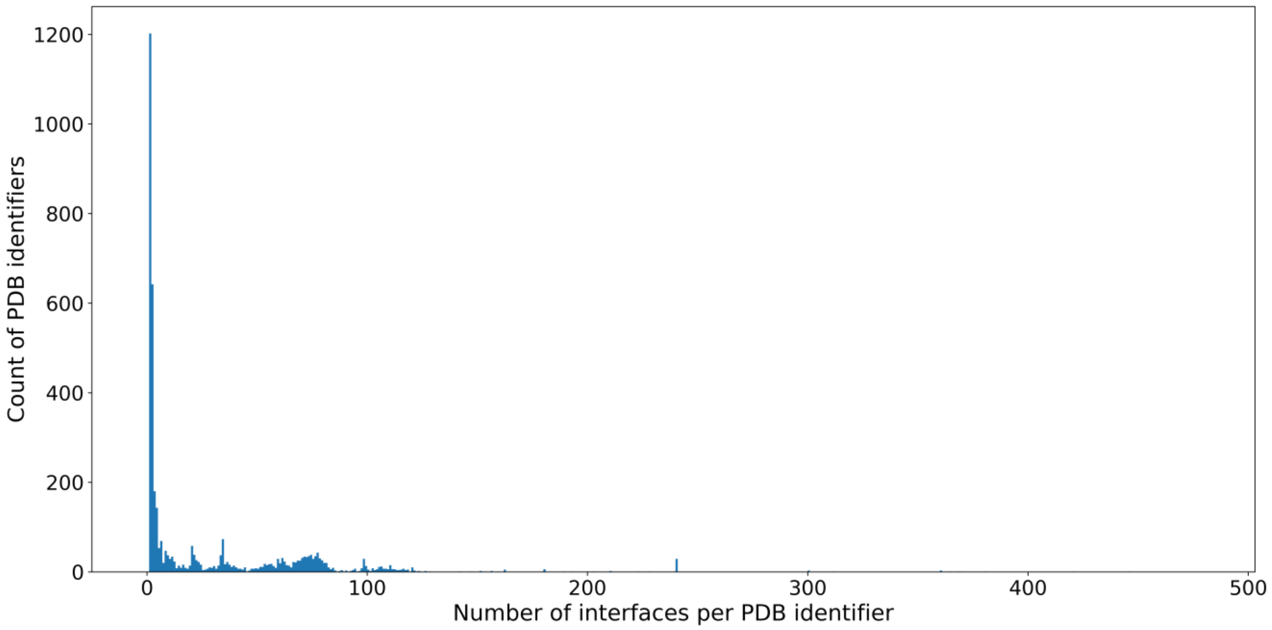

S1 Fig: Histogram representing the number of interfaces per PDB identifier in the initial dataset of 4173 PDB identifiers.
