# Supplementary material for: Structural comparison of homologous protein-RNA interfaces reveals widespread overall conservation contrasted with versatility in polar contacts
Source: PLoS Comput Biol. 2024 Dec 3;20(12):e1012650. doi: 10.1371/journal.pcbi.1012650 (PMC11642956; doi:10.1371/journal.pcbi.1012650)
Supplement: S2 Fig — (PDF) [file pcbi.1012650.s002.pdf]

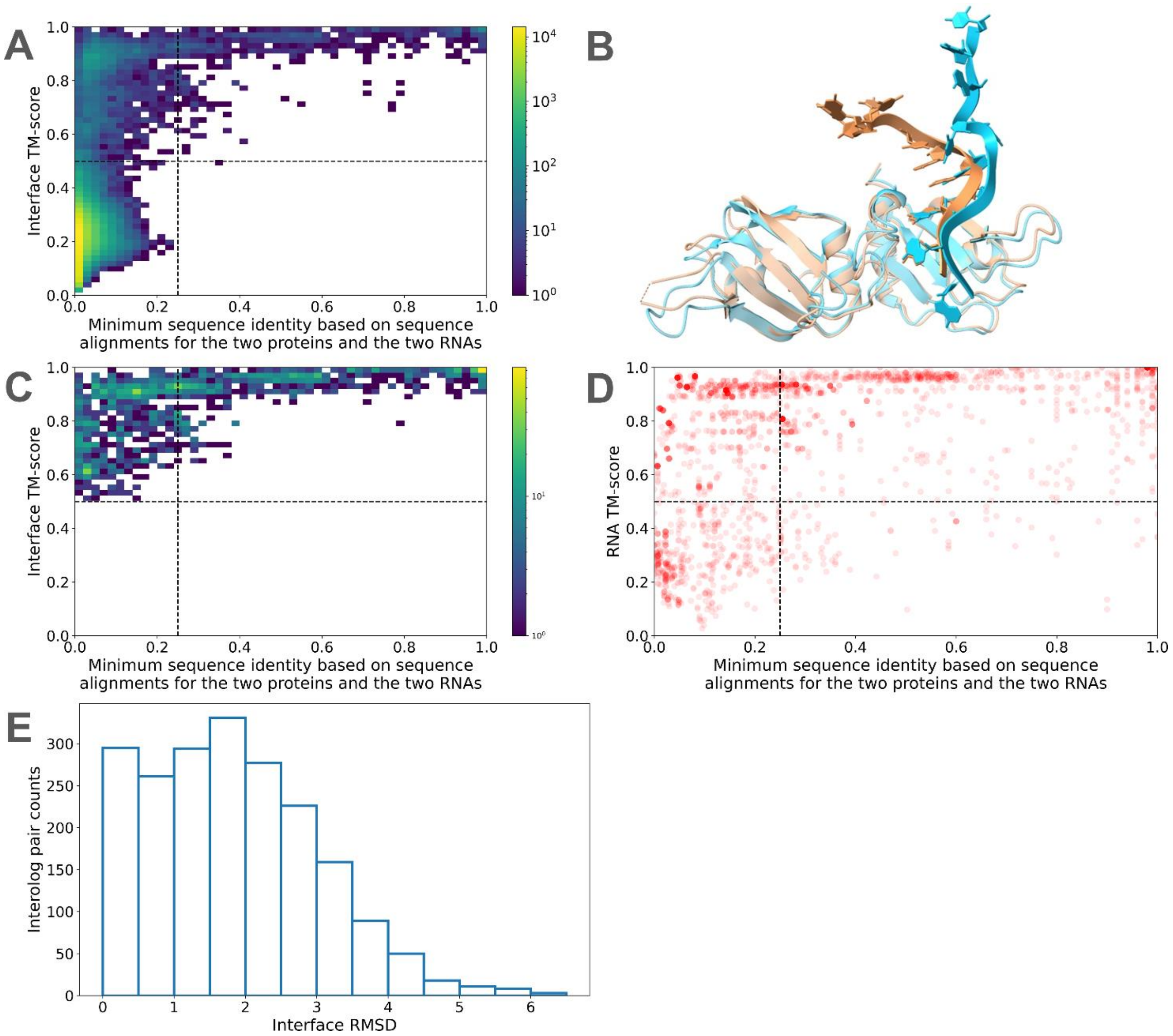

S2 Fig: Supplementary information about the construction of the dataset of interologs. **(A)** 2D histogram of all-against-all interface TM-scores (y-axis) according to (x-axis) the minimum sequence identity within each pair of binary protein-RNA interfaces, weighted by alignment coverage, for 207,326 comparisons (see Fig 2A and 2B). **(B)** Structural alignment of 5HO4_A_B and 5WWE_A_B. For this pair, the protein TM-score is 0.96, the RNA TMscore is 0.13, and the interface TMscore is 0.98. Despite low RNA TM-score (due to the flexibility of the RNA molecule), these interfaces are structural interologs. **(C)** 2D histogram, as in panel A but for the final set of 2,022 interologs (red points in Fig 2B). **(D)** Scatter plot depicting the distribution of RNA TM-scores depending on the minimum interface sequence identity for 2,022 final pairs of confidently assigned structural interologs. 515 pairs have RNA TM-scores below 0.5. This highlights the challenge of using RNA TM-score to define interologs. **(E)** Histogram of interface RMSD (Å) for the 2,022 pairs of interologs. 99.9% have interface RMSD below 6Å and 95.5% have interface RMSD below 4Å.
