# Supplementary material for: Structural comparison of homologous protein-RNA interfaces reveals widespread overall conservation contrasted with versatility in polar contacts
Source: PLoS Comput Biol. 2024 Dec 3;20(12):e1012650. doi: 10.1371/journal.pcbi.1012650 (PMC11642956; doi:10.1371/journal.pcbi.1012650)
Supplement: S3 Fig — (PDF) [file pcbi.1012650.s003.pdf]

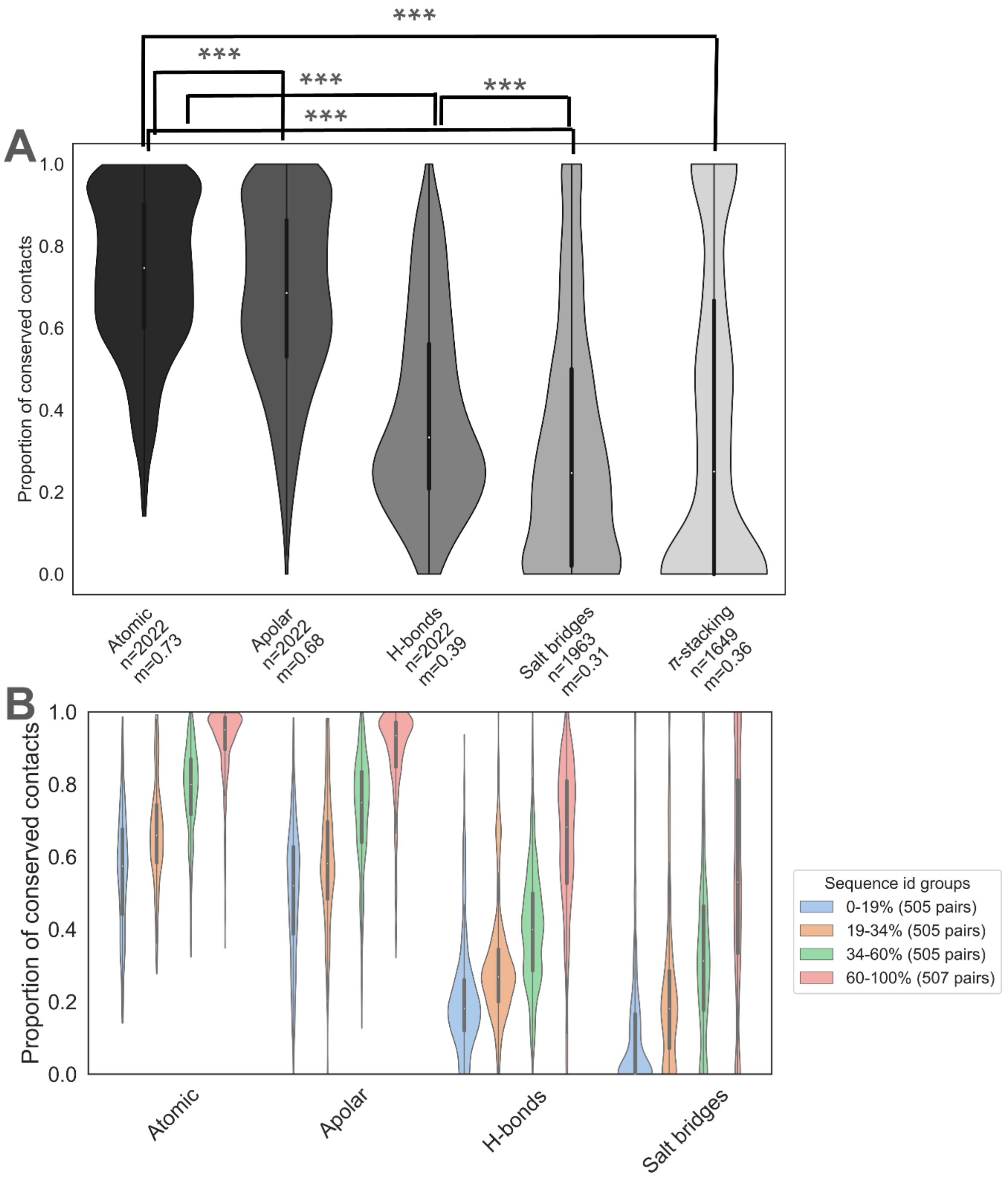

S3 Fig: Supplementary interface contact conservation results. **(A)** Violin plot distributions of contact conservation for distance-based atomic contacts (Atomic), apolar contacts (Apolar), H-bonds, salt bridges and π-stacking. n is the number of interolog pairs used in each violin plot and m is the mean conservation ratio. P-values between distributions denoted by *** in this panel are <3e-38 in a Wilcoxon rank sum test. **(B)** Same as A, but without π-stacking and for pairs of interologs separated into four groups of interface sequence identity (blue: 0-19%, brown: 19-34%, green: 34-60%, red: 60-100%).
