# Supplementary material for: Structural comparison of homologous protein-RNA interfaces reveals widespread overall conservation contrasted with versatility in polar contacts
Source: PLoS Comput Biol. 2024 Dec 3;20(12):e1012650. doi: 10.1371/journal.pcbi.1012650 (PMC11642956; doi:10.1371/journal.pcbi.1012650)
Supplement: S5 Fig — (PDF) [file pcbi.1012650.s005.pdf]

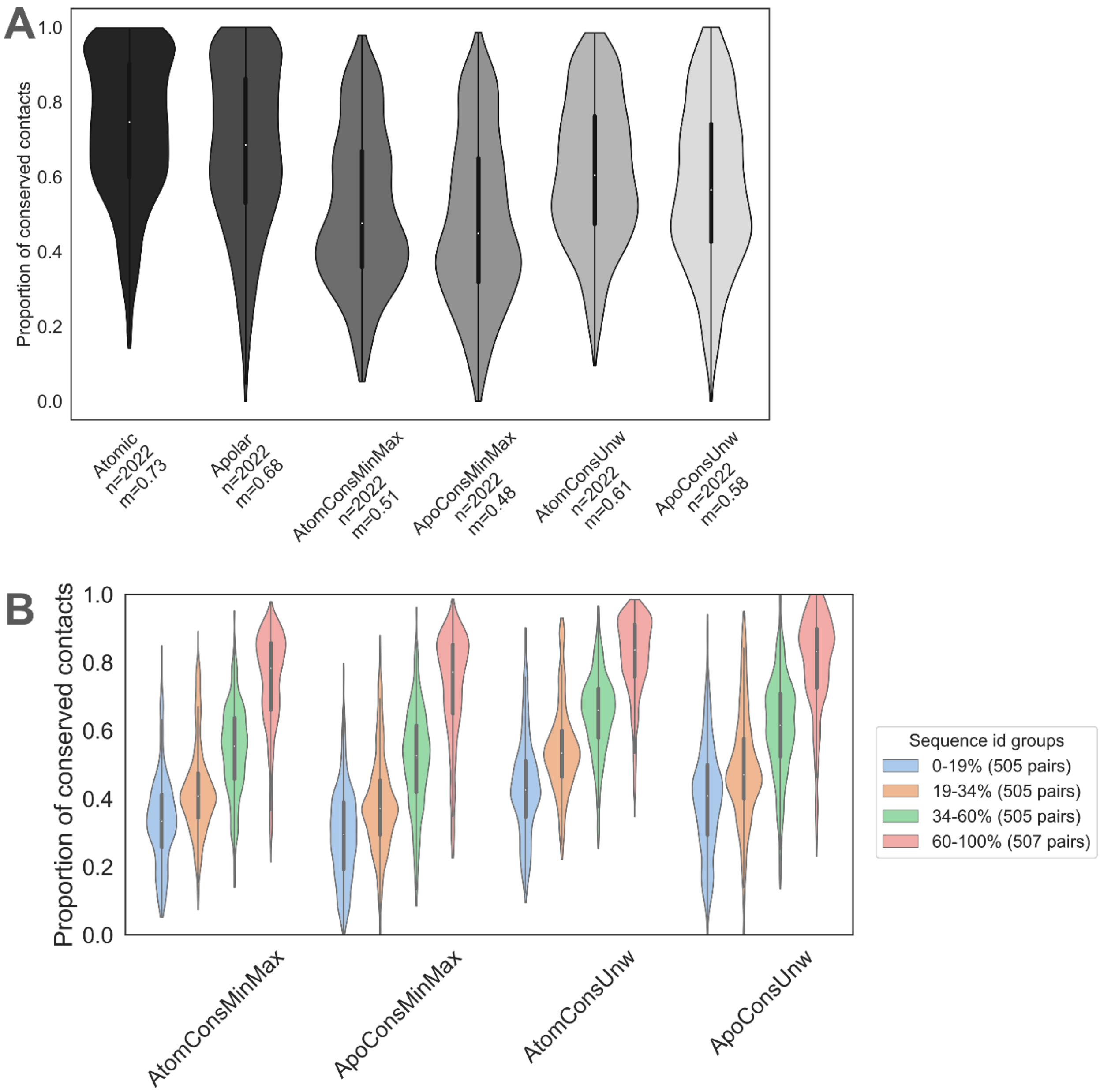

S5 Fig: Distributions of contact conservation using alternative metrics. (A) Overall distributions of AtomicMinMax/ApolarMinMax contact conservation (atomic/apolar conservation calculated with generalized Jaccard index using the ratio of smaller to larger number of atomic contacts in each pair of aligned amino acid-nucleotide contacts), and AtomicNp/ApoNp (non-weighted atomic/apolar contact conservation). The difference between AtomicMinMax and ApolarMinMax conservations is statistically significant (p-value = 9.9e-7 in a Wilcoxon rank sum test). The difference between non-weighted atomic/apolar contact conservation is statistically significant (p-value = 4e-129 in a Wilcoxon rank sum test). (B) Distributions of the same contact conservation values as in panel A, separated into four groups of interface sequence identity (blue: 0-19%, orange: 19-34%, green: 34-60%, red: 60-100%).
