# Supplementary material for: Structural comparison of homologous protein-RNA interfaces reveals widespread overall conservation contrasted with versatility in polar contacts
Source: PLoS Comput Biol. 2024 Dec 3;20(12):e1012650. doi: 10.1371/journal.pcbi.1012650 (PMC11642956; doi:10.1371/journal.pcbi.1012650)
Supplement: S6 Fig — (PDF) [file pcbi.1012650.s006.pdf]

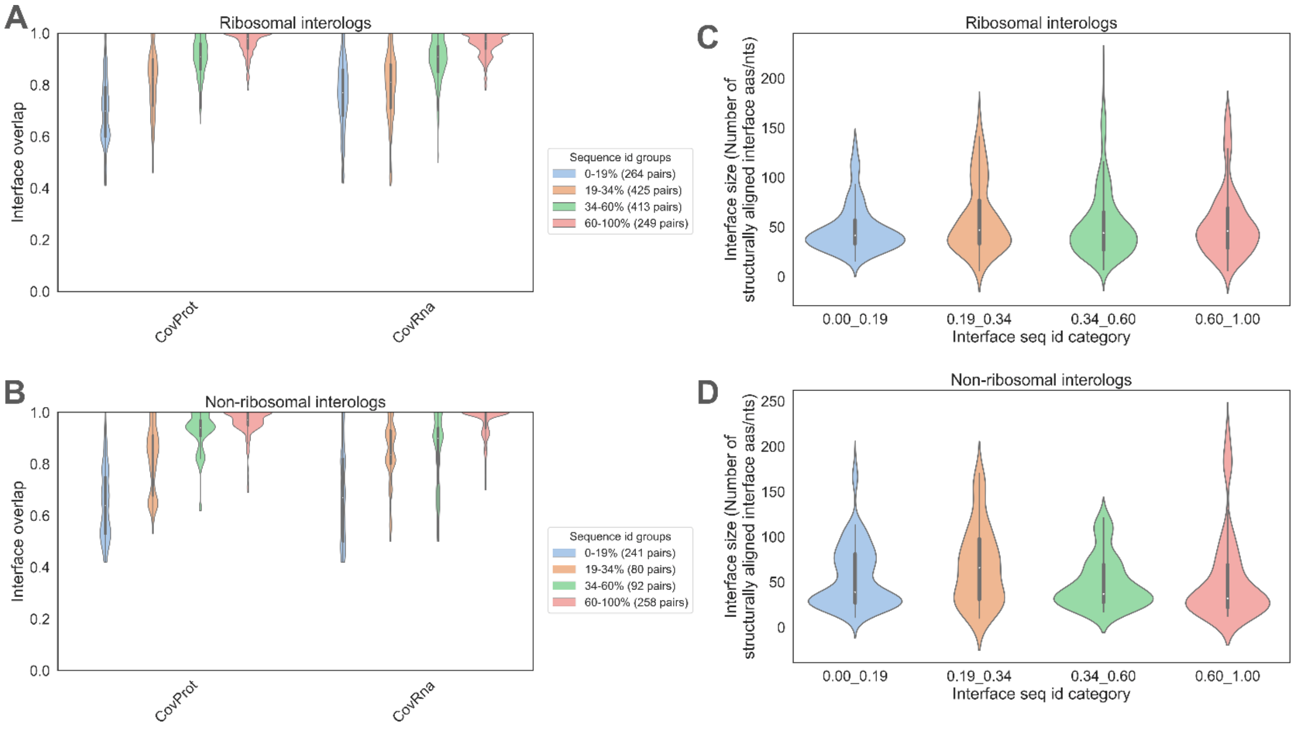

S6 Fig: Interface overlap depending on sequence identity category for ribosomal (panels A, C) and non-ribosomal (panels B, D) interologs. **(A, B)** Violin plots of interface overlap on protein (CovProt) and RNA (CovRna) for each of the 2,022 interolog pairs, separated into four groups of interface sequence identity (blue: 0-19%, orange: 19-34%, green: 34-60%, red: 60-100%). **(C, D)** Violin plots of interface intersect size, measured as the number of structurally aligned interface amino acids/nucleotides for each of the 2,022 interolog pairs, separated into four groups of interface sequence identity (blue: 0-19%, orange: 19-34%, green: 34-60%, red: 60-100%).
