# Supplementary material for: Structural comparison of homologous protein-RNA interfaces reveals widespread overall conservation contrasted with versatility in polar contacts
Source: PLoS Comput Biol. 2024 Dec 3;20(12):e1012650. doi: 10.1371/journal.pcbi.1012650 (PMC11642956; doi:10.1371/journal.pcbi.1012650)
Supplement: S7 Fig — (PDF) [file pcbi.1012650.s007.pdf]

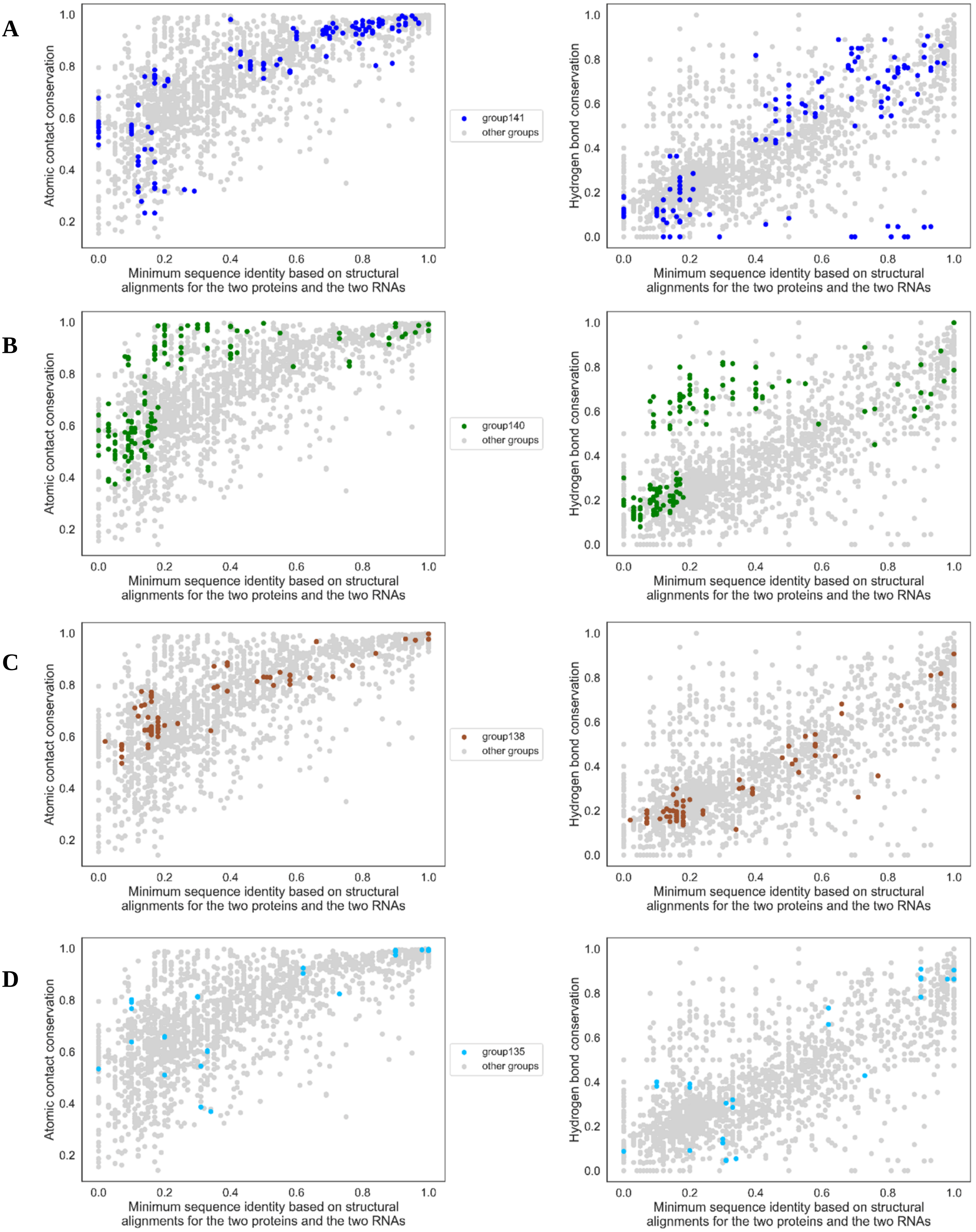

S7 Fig: Distributions of atomic contact conservation (left plots) and H-bond conservation (right plots) depending on interface sequence identity for interolog groups **(A)** g141 (RRM), **(B)** g140 (RNAse H-like including Argonaute), **(C)** g138 (L10e domain with bacterial large ribosomal subunit ribosomal RNA), **(D)** g135 (RNA helicases). On each panel, the colored (as opposed to gray) points represent interologs from the chosen group.
