# Supplementary material for: Structural comparison of homologous protein-RNA interfaces reveals widespread overall conservation contrasted with versatility in polar contacts
Source: PLoS Comput Biol. 2024 Dec 3;20(12):e1012650. doi: 10.1371/journal.pcbi.1012650 (PMC11642956; doi:10.1371/journal.pcbi.1012650)
Supplement: S8 Fig — (PDF) [file pcbi.1012650.s008.pdf]

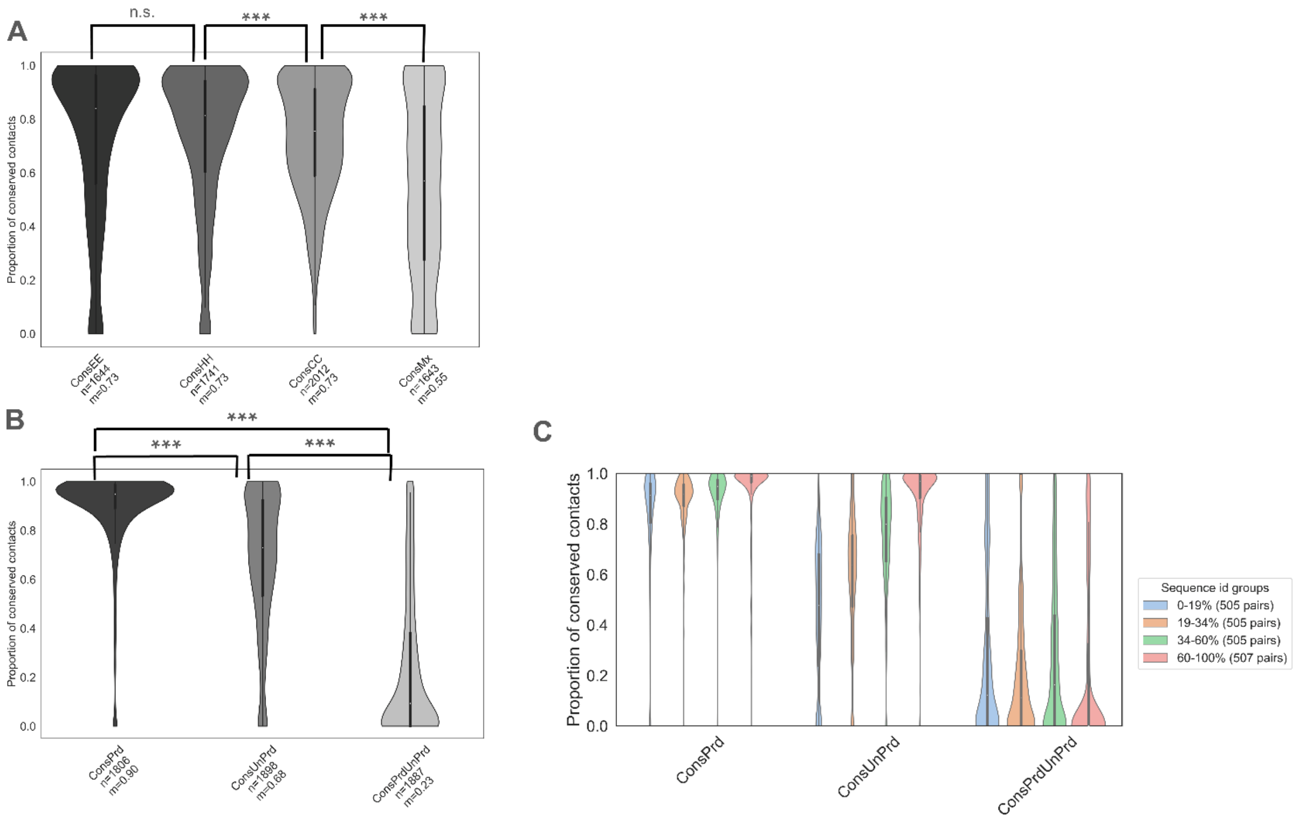

S8 Fig: Violin plots of atomic contact conservation depending on residue secondary structure properties. n is the number of interolog pairs used in each violin plot and m is the mean conservation ratio. **(A)** From left to right with a descending gray palette, distributions of atomic contact conservation where the amino-acid and its structural equivalent keep the same type of secondary structure (strand for ConsEE, coil for ConsCC and helix for ConsHH) or have different secondary structure (ConsMx). P-values between distributions denoted by *** in this panel are <2e-05 in a Wilcoxon rank sum test (and n.s. is not significant). **(B)** From left to right with a descending gray palette, distributions of atomic contact conservation where the nucleotide and its structural equivalent have the same secondary structure (base-paired for ConsPrd and unpaired for ConsUnPrd) or change base-pairing status (ConsPrdUnPrd). P-values between distributions denoted by *** in this panel are <2e-166 in a Wilcoxon rank sum test. **(C)** Distribution of apolar contact conservation according to the same categories as in panel C when splitting the interfaces into four groups of interface sequence identity (blue: 0-19%, orange: 19-34%, green: 34-60%, red: 60-100%).
