# Supplementary material for: Structural comparison of homologous protein-RNA interfaces reveals widespread overall conservation contrasted with versatility in polar contacts
Source: PLoS Comput Biol. 2024 Dec 3;20(12):e1012650. doi: 10.1371/journal.pcbi.1012650 (PMC11642956; doi:10.1371/journal.pcbi.1012650)
Supplement: S9 Fig — (PDF) [file pcbi.1012650.s009.pdf]

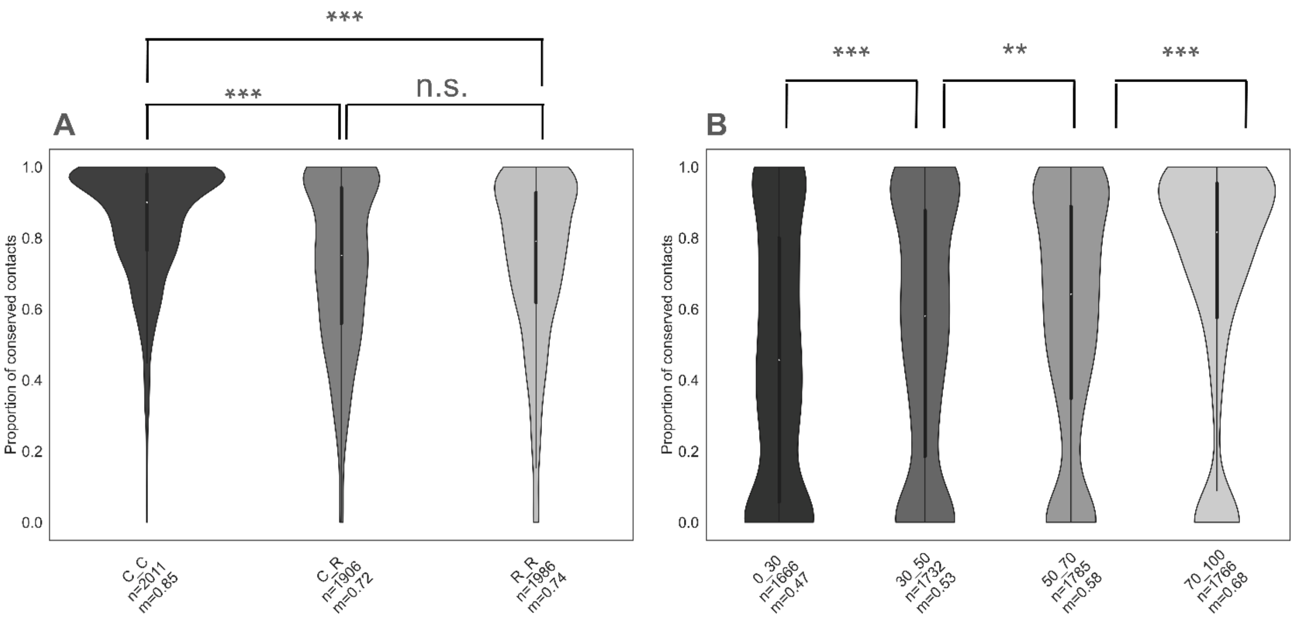

S9 Fig: Violin plots of atomic contact conservation depending on amino acid properties. n is the number of interolog pairs used in each violin plot and m is the mean conservation ratio. **(A)** Violin plot distribution of contact conservation for contacts where amino acids belong to the core of the interface in both interologs (C_C), to core in one interolog and rim in the other interolog (C_R) and to the rim region in both interologs (R_R). The differences between C_C and C_R or R_R are statistically significant (p-value < 4e-56 in Wilcoxon rank sum tests) and the difference between C_R and R_R has p-value 0.16 in a Wilcoxon rank sum test. **(B)** Violin plot distribution of contact conservation. From left to right with for the groups [0:30],]30:50],]50:70],]70:100] respectively according to the minimum value of the evolutionary conservation score within an amino-acid and its structural equivalent. The differences between groups [0:30] and]30:50], and between groups]50:70],]70:100] of conservation scores are statistically significant (p-value < 1.6e-6 in Wilcoxon rank sum tests). Between]30:50], and]50:70], the difference is less significant (p-value = 0.0007 in a Wilcoxon rank sum test).
