# Supplementary material for: Structural comparison of homologous protein-RNA interfaces reveals widespread overall conservation contrasted with versatility in polar contacts
Source: PLoS Comput Biol. 2024 Dec 3;20(12):e1012650. doi: 10.1371/journal.pcbi.1012650 (PMC11642956; doi:10.1371/journal.pcbi.1012650)
Supplement: S10 Fig — (PDF) [file pcbi.1012650.s010.pdf]

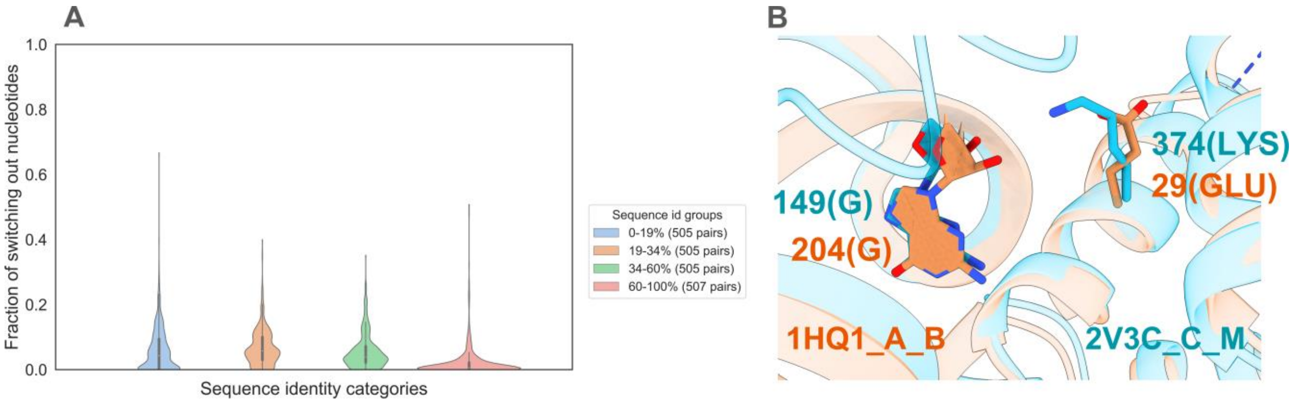

S10 Fig: Supplementary analysis of switching out in contact non-conservation. **(A)** Violin plot distribution of the percentage of switching out nucleotides (weighted by the number of atomic contacts in which each nucleotide is involved) across the four ranges of interface sequence identity. **(B)** Illustration of a residue-nucleotide pair [374(LYS): 149(G)] from interface 2V3C_C_M, for which the corresponding pair in the interolog 1HQ1_A_B is no longer a contact, due to amino acid 29(GLU) switching out of the interface.
