# Supplementary material for: Structural comparison of homologous protein-RNA interfaces reveals widespread overall conservation contrasted with versatility in polar contacts
Source: PLoS Comput Biol. 2024 Dec 3;20(12):e1012650. doi: 10.1371/journal.pcbi.1012650 (PMC11642956; doi:10.1371/journal.pcbi.1012650)
Supplement: S1 Text — (PDF) [file pcbi.1012650.s011.pdf]

Supplementary methods

Identification of ribosomal protein-RNA interfaces

As ribosomal complexes are predominant among protein-RNA complexes in the PDB, we wanted to control ribosome bias in our dataset. We used a list of 400 ribosomal protein identifiers (240 human, including human mitochondrial ribosome proteins, 36 from *Escherichia coli*, and 124 from other species, including *Trypanosoma brucei* and *Saccharomyces cerevisiae*) that cover a large part of our ribosomal dataset, as assessed by a simple regular expression search. We used HHblits and HHsearch [1] version 3.0.0 (15-03-2015) to generate an HMM profile for each of these proteins and search for homologous chains in the pdb70 database [1], which we expanded using the redundancy information (pdb70_clu.tsv file) to cover the entire PDB.

Applying this protocol, 615 of our 977 representative protein-RNA interfaces (63%) were assigned as ribosomal and the remaining 362 as non-ribosomal.

ECOD classification

We assigned ECOD domains at the T-group level (demonstrating homology and similar topological connections) to all interfaces in our dataset. At the interolog group level, we listed and counted all common ECOD domains of interfaces within each group and attributed a most or a set of most represented ECOD T-group domain(s) to this group using the following criteria. If from all ECOD domain labels within a family, there is a single most represented ECOD domain, which is represented in more than 50% of all family members, then the family is given this domain as a label. If several ECOD domains are equally most represented, and they are represented in strictly more than 50% of all family members, then the family is given these domains as a label. If no ECOD domain label is represented in more than 50% of all family members, this family is labeled as "ambiguous". Finally, if there are no ECOD domain labels within a family, then the family is unlabeled.

When comparing pairs of interologs, we look at the intersection between labeled ECOD domains of each interface. In the web interface for database exploration, we assign ECOD labels to groups and pairs of interologs.

Alternative methods of contact conservation calculation

We tested an alternative generalized Jaccard index [2, 3]. In this setting, contact conservation was determined for a given pair of interologs by calculating the ratio of the sum of the minimum number of atomic contacts over all aligned amino acid-nucleotide pairs divided by the sum of the maximum number of atomic contacts. The contact conservation distribution was then assessed for each pair of interologs in the dataset.

We also computed a non-weighted version of conservation where the calculation does not involve weighting based on contact numbers. Instead, it focuses solely on the presence or absence of contact (defined by heavy-atom minimum distance of 5 Å) in each interface residue-nucleotide pair and its structural equivalent for each pair of interologs.

Random baselines for contact conservation

Some contacts are more frequent than others: in particular, atomic and apolar contacts are much more frequent than hydrogen bonds, salt bridges and stacking, and they can be made more easily by any kind of amino acid, while polar contacts and stacking require specific properties of amino acid side chains. Therefore, it is legitimate to wonder to what extent the levels of contact conservation we observe between interologs can be driven by chance, as a neighboring amino acid (aa) and nucleotide (nt) will make atomic contacts, and in most cases apolar contacts.

We designed two random baselines to address this question. In both baselines, we maintain the "scaffold" of the interface, i.e. which protein/RNA positions are part of this interface (thus, switching out is unaffected in the baselines), and the distances between the CA atom of protein positions and

the C3' atom in RNA positions, and we reassign the aa and nt type, as well as the interface aa/nt contacts. In both baselines, interface aa/nt contacts are drawn from the distribution of original interface contacts (in all 765 interfaces from our dataset). The drawn contacts depend on the (re-assigned) aa and nt nature and the (unchanged) binned distance between the CA atom (in protein) and the C3' atom (in RNA). This allows us to model how many atomic/apolar contacts the aa/nt pair will form, and whether it will form H-bonds, salt bridges, or stacking contacts. For instance, a pair between Arg and U at a binned CA-C3' distance of 16 to 18 Å will get assigned a number of atomic contacts and apolar contacts, and a status for H-bonding, salt bridge and stacking (True or False), drawn from the original distribution of all Arg/U pairs at CA-C3' distance of 16 to 18 Å. By construction, the number of apolar contacts is smaller than the number of atomic contacts, and there is an interdependency between H-bond/stacking and atomic contacts and between H-bonds and salt bridges. Note that the drawn number of atomic/apolar contacts can be zero, especially if the CA-C3' distance is large. Based on observed contact distributions, we defined the following CA-C3' distance bins: 0-6 Å (in practice, 3-6 Å), 6-8 Å, 8-10 Å, 10-12 Å, 12-14 Å, 14-16 Å, 16-18 Å and 18-20 Å. Beyond a CA-C3' distance of 20 Å, we assign no more contacts to this pair.

The first baseline ("shuffled") is a random shuffling of interface residue types. This baseline maintains the amino acid composition of the interface; however, it greatly reduces the sequence identity between interologs (to levels close to random i.e. around 7% for proteins and 23% for RNA). Therefore, we designed a second baseline ("resampled") where the interface is resampled from a distribution of amino acids/nucleotides following overall protein-RNA interface frequencies (e.g. Cys and Trp are much less frequent than Arg or Lys). When resampling, we set a bias towards drawing the same aa/nt as in the original interface, in order to maintain interface sequence identity. Note that in this resampled baseline, even when the aa/nt type is the same as in the original interface, the contacts they make are reassigned by drawing from the previously described distribution.

In both baselines, we re-assess contact conservation by keeping one interolog from the original dataset and using the shuffled/resampled version for the second interolog.

Note that in both baselines, we exclude contacts involving non-canonical aa/nt types, and all aa/nt without CA/C3' atom coordinates.

Supplementary results

Initial PDB dataset

Many of the initial 4173 PDB identifiers contain a large number of protein and RNA chains; therefore, the average number of protein-RNA interfaces per PDB identifier in our initial dataset (Fig 1) is around 28. However, this reflects a diverse range of situations, with 1202 PDB identifiers containing a single interface, and only 218 PDB identifiers with more than 100 interfaces each (see distribution in S1 Fig).

Interface analysis and composition

Resulting from the 977 all against all structural alignment, we obtained 2,022 interologs from 765 interfaces. We analyzed this dataset of 765 interfaces after checking that no major differences were observed compared to the dataset of 977 interfaces. Among the 765 interfaces, 33% of the interacting amino acids are in a helix, 10% in a β-strand, and 57% in a "coil" region. Regarding nucleic acid secondary structure, 75% of nucleotides were paired, and 25% were unpaired.

We also assessed the distribution of interface contacts according to the assignment of core/rim protein interface regions: in 55% of the contacts, the amino acid belongs to the core region and in 45% to the rim region.

We analyzed the global number of atomic contacts for each interface. We found that, on average, an interface contains 1160 atomic contacts (vs. 230 for protein-protein interfaces) (1235 for ribosomal

interfaces, 987 for non-ribosomal interfaces) and 257 apolar contacts (262 for ribosomal interfaces, 243 for non-ribosomal interfaces). When grouped into residue-nucleotide contacts, an interface has an average of 109 distance-based contacts (120 for ribosomal interfaces, 89 for non-ribosomal interfaces), much more than for protein-protein interfaces, on which the average was 61 [4]. Each interface has on average 2 π-stacking interactions (2 for ribosomal interfaces, 3 for non-ribosomal interfaces) and 30 hydrogen bonds (33 for ribosomal interfaces, 23 for non-ribosomal interfaces). The higher number of stacking interactions in the non-ribosomal interfaces may be explained by different residue type compositions, particularly aromatic amino acids representing 11% of the amino acids in the non-ribosomal interfaces vs. 7% in ribosomal interfaces. We verified that the composition of the interface is consistent with previous statistical analyses performed on smaller sets of heteromeric protein-RNA complexes [5].

Previous studies highlighted that the backbone phosphates are involved in the majority of protein-RNA interactions [6]. It is also the case in our contact dataset, where 59% of the hydrogen bonds involve the phosphate group of the nucleotide. 76% of the amino acids performing an H-bond involve their side chains.

Supplementary interologs analysis

Interologs were split into ribosomal and non-ribosomal: interologs were assigned as ribosomal if both protein chains in the structural interolog pairs were assigned as ribosomal protein chains and non-ribosomal when the two protein chains were assigned as non-ribosomal. We decided not to include interologs where one interface was assigned as ribosomal and the other as non-ribosomal; these pairs represented only 2% of our interologs pairs.

Applying these criteria led to the identification of 2,022 interologs, out of which 1371 were ribosomal and 671 non-ribosomal. Those interologs come from 765 interfaces, and those interfaces are made from 257 PDB complexes. 176 (69%) have resolution higher than 2Å.

Among the 2,022 interolog pairs 48 have both interface resolutions lower than 2Å, and in 463 cases, one structural interolog within the pair has resolution lower than 2Å and the other higher than 2Å. In the remaining 1391 cases, both have resolutions higher than 2Å.

The numbers of contacts differ by 19% on average between two interologs for atomic contacts (vs. 15% for protein-protein interfaces) and by 27% for apolar contacts.

Interologs are separated into four equally populated groups of interface sequence identity: the 0-19%, 19-34%, and 34-60% sequence identity groups each contain 505 pairs of interologs, while the 60-100% sequence identity group contains 507 pairs of interologs.

When counting the number of structurally aligned amino acid-nucleotide pairs, where the pair is at a distance shorter than 5 Å in at least one of the two interologs, the 0-19% group has 43342 such pairs, while the 19-34%, 34-60%, and 60-100% have respectively 69337, 62435 and 61786. Hence, the contact conservation in the 0-19% group might be moderately over-estimated because many amino acid-nucleotide pairs cannot be structurally aligned.

Supplementary contact conservation analysis

To verify that the observed trends were not due to our way of weighing the conservation metrics, we also analyzed distributions of contact conservation computed with two alternative metrics, either unweighted (i.e. where each amino acid-nucleotide pair counts for 1) or following an alternative definition of generalized Jaccard index (using the ratio of smaller to larger number of atomic contacts in each pair of aligned amino acid-nucleotide contacts, see supplementary methods above).

Despite the values of conservation being overall lower with these alternative metrics, we observed similar trends of increasing contact conservation with increasing interface sequence identity (S2 Fig).

With the alternative generalized Jaccard index, apolar contacts appear slightly less conserved than atomic contacts (S2 Fig). This can be explained by the observation that within a pair of aligned amino acid-nucleotide contacts, the numbers of atomic contacts are more similar than the numbers of apolar contacts (difference of 20% vs. 28% on average); therefore the minimum and maximum numbers of atomic contacts are more similar than the minimum and maximum numbers of apolar contacts and the min/max ratio will be closer to 1 for atomic contacts.

Apart from S2 Fig, we performed all analyses in the present study using the Jaccard index weighted by the number of atomic contact pairs.

The contact conservation is, on average, 22% when the nucleotide changes base-pairing status between one interolog and the other. To quantify this observation, we analyzed the subgroup of contacts where the nucleotide is base-paired in one interolog and unpaired in the other, constituting 30% of the pairwise contacts. Among this subgroup's 62,182 non-conserved pairwise contacts, 62,032 (99%) are attributed to contacts involving base-paired nucleotides that were lost when the nucleotides transitioned to an unpaired state.

Random baselines for contact conservation

The contact conservation levels are similar in both baselines (see supplementary methods above): on average 54-55% for atomic contacts, 36-37% for apolar contacts, 9-11% for H-bonds, 2-4% for salt bridges, 0-2% for stacking. These numbers are largely smaller than average conservation levels in the original interolog dataset, respectively, 73% for atomic contacts, 68% for apolar contacts, 39% for H-bonds, 31% for salt bridges and 36% for stacking.

The contact conservation in both baselines shows a small dependency on the original sequence identity category of the interolog pair (i.e. before shuffling/resampling), even in the shuffled dataset. This translates an increasing correlation between CA-C3’ distances of aligned aa/nt pairs with

increasing sequence identity (our baselines do not change the CA-C3' distances, only the aa/nt nature and the exact contacts they make): the Pearson correlation between CA-C3' distances of aligned aa/nt pairs is 0.71 for sequence identity category 0-19%, 0.77 for 19-34%, 0.90 for 34-60%, and 0.97 for 60-100%.

The difference in contact conservation levels between the original and baseline datasets is also large when considering each sequence identity category. The contact conservation levels in both baselines are very similar for ribosomal and non-ribosomal interologs.

The smallest relative difference occurs for atomic contacts, highlighting that despite their overall higher conservation compared to other contact types, they are probably the least specifically conserved in interologs.

Interolog groups

In group 141 (RRM family), interface 2NZ4_A_P (X-ray structure at 2.5 Å resolution) is responsible for all the points at very low H-bond conservation despite high sequence identity and high atomic contact conservation (S7 Fig, top-most panels, consider the blue points at the bottom right of the right-side H-bond conservation plot). The resolution of this structure seems to be insufficient to correctly position atoms in order to follow H-bond forming geometric criteria. This is a good illustration of the necessity to choose high-resolution structures to perform in-depth analysis of H-bond conservation in our study.

References

1. Remmert M, Biegert A, Hauser A, Soding J. HHblits: lightning-fast iterative protein sequence searching by HMM-HMM alignment. Nat Methods. 2011;9(2):173-5. doi: 10.1038/nmeth.1818.

2. Chierichetti F, Kumar R, Pandey S, Vassilvitskii S. Finding the Jaccard Median. Proc Appl Math. 2010;135:293-311. PubMed PMID: WOS:000280699900025.

3. Ioffe S, editor Improved Consistent Sampling, Weighted Minhash and L1 Sketching. 2010 IEEE International Conference on Data Mining; 2010 13-17 Dec. 2010.

4. Andreani J, Faure G, Guerois R. Versatility and invariance in the evolution of homologous heteromeric interfaces. PLoS Comput Biol. 2012;8(8):e1002677. doi: 10.1371/journal.pcbi.1002677.

5. Treger M, Westhof E. Statistical analysis of atomic contacts at RNA-protein interfaces. Journal of molecular recognition: JMR. 2001;14(4):199-214. doi: 10.1002/jmr.534.

6. Kagra D, Jangra R, Sharma P. Exploring the Nature of Hydrogen Bonding between RNA and Proteins: A Comprehensive Analysis of RNA : Protein Complexes. ChemPhysChem. 2022;23(2):e202100731. doi: 10.1002/cphc.202100731.
