# Supplementary material for: Structural comparison of homologous protein-RNA interfaces reveals widespread overall conservation contrasted with versatility in polar contacts
Source: PLoS Comput Biol. 2024 Dec 3;20(12):e1012650. doi: 10.1371/journal.pcbi.1012650 (PMC11642956; doi:10.1371/journal.pcbi.1012650)
Supplement: S2 Text — (PDF) [file pcbi.1012650.s012.pdf]

**Seq id category**	**0-19%**		**19-34%**		**34-60%**		**60-100%**	
Rib/non-rib interologs	rib	non-rib	rib	non-rib	rib	non-rib	rib	non-rib
Atomic	0.54	0.58	0.65	0.74	0.76	0.87	0.90	0.93
	p-value: 0.2		p-value: 3e-5		p-value: 5e-15		p-value: 3e-5	
Apolar	0.46	0.54	0.57	0.67	0.70	0.83	0.86	0.92
	p-value: 5e-6		p-value: 3e-4		p-value: 2e-14		p-value: 1e-8	
H-bonds	0.17	0.25	0.28	0.43	0.38	0.53	0.61	0.68
	p-value: 2e-5		p-value: 4e-9		p-value: 4e-15		p-value: 3e-5	
Salt bridges	0.09	0.16	0.20	0.36	0.30	0.45	0.55	0.55
	p-value: 0.05		p-value: 4e-4		p-value: 2e-4		p-value: 0.6	

Table A: Average values of contact conservation, separately for each sequence identity category and ribosomal/non-ribosomal interologs. The indicated p-values correspond to ribosomal vs. non-ribosomal distributions in a Wilcoxon rank sum test.

**Seq id category**	**0-19%**		**19-34%**		**34-60%**		**60-100%**	
Rib/non-rib interologs	rib	non-rib	rib	non-rib	rib	non-rib	rib	non-rib
Median seq id	0.14	0.10	0.24	0.25	0.45	0.48	0.77	0.87
Mean seq id	0.12	0.10	0.25	0.26	0.46	0.48	0.80	0.86

Table B: Median and mean values of sequence identity, separately for each sequence identity category and ribosomal/non-ribosomal interologs.
