# Supplementary material for: Structural comparison of homologous protein-RNA interfaces reveals widespread overall conservation contrasted with versatility in polar contacts
Source: PLoS Comput Biol. 2024 Dec 3;20(12):e1012650. doi: 10.1371/journal.pcbi.1012650 (PMC11642956; doi:10.1371/journal.pcbi.1012650)
Supplement: Attachment — Submitted filename: 202409_ploscomp_response-to-reviewers_R1.pdf [file pcbi.1012650.s013.pdf]

We thank the editors and all three reviewers for their assessment and suggestions. Please find a point-by-point response below (in blue). We made the corresponding changes in the main text (line numbers highlighted in green), figures, supplementary material. Whenever relevant, we also carried over the changes into the accompanying web interface and Jupyter notebook.

Reviewer #1: The paper addresses an important problem of protein-RNA interactions from structural, physicochemical and evolutionary perspective. The protein-RNA interactions play a key role in cellular mechanisms. Our ability to understand, model and manipulate these interactions to reveal biological mechanisms and for drug design is critically dependent on systematic studies of protein-RNA interfaces. Currently, such studies and the related modeling approaches significantly lag behind those focusing on protein-protein interactions. Advances in deep learning applications, such as AlphaFold3, do not negate but rather re-emphasize the need for protein-RNA knowledge resources. The paper in a significant way contribute to filling this knowledge gap. The authors provide a systematic analysis of the existing data on protein-RNA interactions, with great attention to details and interpretation of the results. The authors are experts on the subject. The paper is well written and illustrated. The related public resources will be of great use for protein-RNA studies. As such, the paper is of significant interest to the research community.

We thank Reviewer #1 for their very enthusiastic comments and their appreciation of our work.

Reviewer #2: Mahmoudi et al present an analysis of the structural conservation of protein-RNA complexes across the PDB. To do so they first derive a dataset of 977 non-redundant protein-RNA complexes (representative interfaces), and clustered 765 of these into 2022 (interolog) pairs of homologous protein-RNA complexes. They show that apolar contacts are relatively well conserved across interologs, while H-bridges, salt bridges and pi-stacking contacts are poorly conserved, sometimes even across interologs with high sequence identity. They also show that the non-conservation of atomic contacts between interologs is often driven by what they call "switching-out" of the interface, mainly of the amino-acid making the contact, or by the nucleotide. The work is an interesting and valuable description/analysis of protein-RNA interactions.

The manuscript is generally well written and presented, although the clarity of some aspects could be improved (detailed below). Certain aspects of the analysis could also be improved/added, in particular regarding the contribution of ribosomal protein-RNA complexes in the dataset. This is a major point as it is now unclear whether this work describes mostly trends related to ribosomal protein-RNA complexes, or whether it describes protein-RNA complexes in general. The results on the conservation of certain contact types could also be improved by providing an expected "baseline".

We thank Reviewer #2 for their overall very positive assessment of our work. We provide point-by-point replies to the detailed comments below (including ribosomal vs. non-ribosomal analysis and conservation "baseline").

General comments (we numbered the points for an easier response)

1- I don't understand how the interolog's structure shown in Fig 2C passed the first filtering steps. The TM-score for the two proteins is 0.74 because normalized by the smallest structure, but the coverage appears very low (the two RNA structures don't appear to bind on the same

surface site, so how could it pass the initial thresholds?) - I don't understand how it passed the filtering step "MM-align succeeds in aligning protein and RNA in interface", nor the filtering step on interface TM-score > 0.5

We chose this (non-interolog) example precisely to illustrate some limitations in MM-align interface alignment. In Fig 2A, “MM-align succeeds in aligning protein and RNA in interface” represents the ability of MM-align to align simultaneously the protein chains and the RNA chains and to produce an interface TM-score. However, when MM-align is able to align two interfaces, the interface TM-score is not always a reliable metric. The 5AOR_A_C; 3ADL_A_B,C example is representative of a good MM-align interface TM-score despite no interface similarity (as the reviewer correctly notes, the two RNAs do not bind on the same protein region). A server run of MM-align reproduces this behavior (as jobs are kept for a few days only on the server, please find a screenshot below). Even though in this case, normalization by the smallest interface would yield an interface TM-score < 0.5, we choose normalization by the smallest structure for TM-scores, because many of our comparisons involve complexes of very different sizes, and other normalizations would miss meaningful structural alignments.

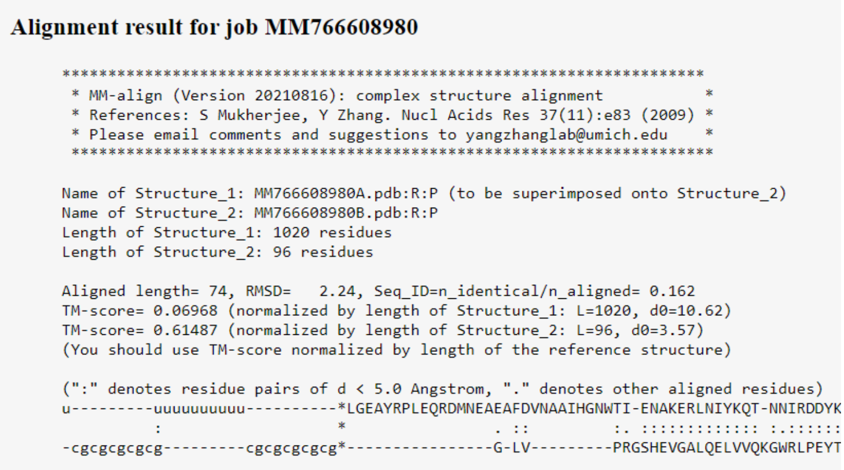

Alignment result for job MM766608980

```
 *************************************************************************
  * MM-align (Version 20210816): complex structure alignment             *
  * References: S Mukherjee, Y Zhang. Nucl Acids Res 37(11):e83 (2009) *
  * Please email comments and suggestions to yangzhanglab@umich.edu    *
  *************************************************************************

 Name of Structure_1: MM766608980A.pdb:R:P (to be superimposed onto Structure_2)
 Name of Structure_2: MM766608980B.pdb:R:P
 Length of Structure_1: 1020 residues
 Length of Structure_2: 96 residues

 Aligned length= 74, RMSD=   2.24, Seq_ID=n_identical/n_aligned= 0.162
 TM-score= 0.06968 (normalized by length of Structure_1: L=1020, d0=10.62)
 TM-score= 0.61487 (normalized by length of Structure_2: L=96, d0=3.57)
 (You should use TM-score normalized by length of the reference structure)

 (":" denotes residue pairs of d < 5.0 Angstrom, "." denotes other aligned residues)
 u---------uuuuuuuuuu----------*LGEAYRPLEQRDMNEAEAFDVNAAIHGNWTI-ENAKERLNIYKQT-NNIRDDYK
           :                   *                .  ::          :. ::::::::::::: :.:::::
 -cgcgcgcgcg---------cgcgcgcgcg*----------------G-LV---------PRGSHEVGALQELVVQKGWRLPEYT
```

The 5AOR_A_C; 3ADL_A_B, C pair is therefore included in the 6,479 pairs of Fig 2A (after filtering for interface TM-score > 0.5) even though the two interfaces are actually not similar. This is why we defined additional filters based on interface overlap on both the protein and RNA sides, and protein TM-score. The 5AOR_A_C; 3ADL_A_B, C pair fails the interface overlap filter, and is therefore not included in the final 2,022 pairs.

We clarified the legend of **Fig 2C** (lines 206-211): “An example pair of non-interolog interfaces aligned with MM-align where the interface TM-score is above 0.5 despite low interface coverage. 5AOR_A_C; 3ADL_A_B, C belongs to the intermediate set of 6,479 pairs. The two RNA chains do not bind in the same protein region so that the RNA interface overlap is 0% and this pair of interfaces is not retained in the final set of 2,022 structural interologs.” We also clarified the accompanying text (line 184): “we observed that interfaces may not be similar even when the interface TM-score provided by MM-align is high, e.g. because of low interface overlap”.

2- The previous comment involves the calculation of an “interface TM-score” but I could not find a description in the methods. This should be described in detail.

The interface TM-score is provided directly by MM-align as the main metric to assess interface similarity. We added this information on line 174 (i.e. the first time the interface TM-score is mentioned in the results: "the interface TM-score provided by MM-align") and we rephrased the sentence stating this in the Methods to clarify this point (last sentence in "Structural alignment" section, lines 665-669):
"For each pair of compared interfaces, we used the protein, RNA, and interface TM-scores provided by each software tool (respectively TM-align, RNA-align and MM-align). The software provides TM-scores normalized by each molecule/interface separately; we retained only the TM-scores normalized by the smallest molecule/interface, as many of our comparisons involve complexes of very different sizes."

3- The authors wrote: "Distance-based contacts are also much more conserved compared to what we had found in a previous study of homologous protein-protein interfaces. In contrast, hydrogen bonds, salt bridges, and π-stacking interactions are very versatile in pairs of protein-RNA interologs, even for close homologs with high interface sequence identity." An important conclusion of the work is that certain contact types are more conserved than others, in particular apolar contacts appear more conserved than others. At the same time, because apolar contacts are much more frequent to begin with, a random reshuffling of contacts will naturally result in apolar contact being frequent and others being rare. For that reason, this observation should be compared to a carefully crafted random baseline.

We thank the reviewer for raising this important point. Indeed, some contacts are more frequent than others, as we already noted in the initial manuscript. In particular, atomic and apolar contacts are much more frequent than hydrogen bonds, salt bridges and stacking, and they can be made more easily by any kind of amino acid, while polar contacts and stacking require specific properties of amino acid side chains. Therefore, it is legitimate to wonder to what extent the levels of contact conservation we observe between interologs can be driven by chance, as neighboring amino acids and nucleotides will make atomic contacts and in most cases apolar contacts.

We designed two random baselines to address this question. The first one, as suggested by the reviewer, is a random reshuffling of interface residues followed by re-assessment of contact conservation. This baseline maintains the amino acid composition of the interface; however, it greatly reduces the sequence identity between interologs (to levels close to random i.e. around 7% for proteins and 23% for RNA). Therefore, we designed a second baseline where the interface is "resampled", i.e. the type of each interface residue is drawn from a distribution of amino acids/nucleotides (following overall interface frequencies, e.g. Cys and Trp are much less frequent than Arg or Lys). We set a bias towards drawing the same aa/nt as in the original interface, to maintain interface sequence identity.

In both baselines, we maintain the "scaffold" of the interface, i.e. which protein/RNA positions are part of this interface and the distances between the CA atom of protein positions and the C3' atom in RNA positions, and we reassign the aa and nt type, as well as the interface aa/nt contacts. Interface aa/nt contacts are drawn from the distribution of original interface contacts (in all 765 interfaces from our dataset). The drawn contacts depend on the (re-assigned) aa and nt nature and the binned distance between the CA atom (in protein) and the C3' atom (in RNA). This allows us to model how many atomic/apolar contacts the aa/nt pair will form, and whether it will form H-bonds, salt bridges, or stacking contacts.

The contact conservation levels are similar in both baselines, and largely smaller than average conservation levels in the original interolog dataset. The smallest relative difference occurs for atomic contacts, highlighting that despite their overall higher conservation compared to other contact types, they are probably the least specifically conserved in interologs.

The contact conservation in both baselines shows a small dependency on the original sequence identity category of the interolog pair (i.e. before reshuffling/resampling), even in the reshuffled dataset. This translates an increasing correlation between CA-C3' distances of aligned aa/nt pairs with increasing sequence identity (which we now report about in the Supplementary results).

We included these random baselines in the Supplementary methods and results and we added a corresponding Supplementary **Fig S4**. We also added them in the results section, on lines 261-273 (overall contact conservation), 312-313 (ribosomal vs non-ribosomal interologs) and 324-327 (influence of interface sequence identity).

4- l.281 An important point is to give precise numbers on how many of those protein-RNA complexes are part of ribosomal complexes. Out of the final 2022 pairs, how many of these are related to ribosomes? Some key figures on statistics (e.g. Fig. 2D) could be provided for both types separately in the sup. mat.

We agree that this is an important question and we had already provided some information in the supplementary results: "2,022 interologs, out of which 1371 were ribosomal and 671 non-ribosomal" and supplementary Fig S3 where contact conservation (as shown in original Fig 3C/3D for the whole dataset) was split between ribosomal and non-ribosomal interologs.

Following also the related comment (#1) of Reviewer #3, we investigated the ribosomal vs. non-ribosomal behavior in more detail and we updated Fig 3 (now **Fig 4**) to reflect the distributions for these two categories of interfaces. We moved overall distributions to supplementary **Fig S3**. We updated and reorganized the manuscript text to accommodate the deeper focus given to ribosomal/non-ribosomal interface tendencies (lines 280-284 and 304-313).

5- Fig. 1: It is not clear how we go from 4173 complexes to 115085 interfaces. Does this mean there are ~30 interfaces per PDB file, on average? This could be described.

Exactly. Many of the 4173 PDB identifiers contain a large number of protein and RNA chains; therefore indeed, the average number of protein-RNA interfaces per PDB identifier in our initial dataset is around 28. However, this reflects a diverse range of situations, with 1202 PDB identifiers containing a single interface, and 218 PDB identifiers with more than 100 interfaces each. The text was updated to describe this (on lines 148-150) and refer to an additional paragraph in supplementary results and additional supplementary **Fig S1** showing the distribution of the number of interfaces per PDB identifier.

6- Fig. 1: after filtering, only 3383 interfaces are retained. Why are so many interfaces filtered out? Is it due to RNA in contact being too small? In that case, should have it been considered as protein-RNA complex in the first place? This should also be described, even if with just one sentence.

The resolution filter (i.e. removing NMR structures, as well as X-ray and cryo-EM structures with resolution > 2.5 Å) explains a large majority of the 115,085 interfaces that are filtered out. The text was updated to described this (on lines 151-152) and **Fig 1** was also updated to include an intermediate step where 6,369 interfaces remain after resolution filtering. We then proceed to size filtering, yielding 3,419 interfaces, then to clustering, yielding the representative dataset of 977 interfaces.

(Please note that the exact numbers in Fig 1 were corrected, with very slight changes, so that they now match the provided Jupyter notebook and data files).

7- Fig 2B should also highlight the final set of 2022 pairs (with a different color for instance). This set is more important to highlight than the 6079 pairs, which are the only ones shown currently. I would also suggest plotting a contour density for the 3 sets rather than using points are those obscur the density completely.

The 6,079 pairs are no longer highlighted, as they simply correspond to all points above the horizontal line plotted at interface TM-score = 0.5. Instead, red dots represent the 2,022 pairs (we chose this option over a third color, for better plot clarity). We added partial transparency to the scatterplot in **Fig 2B** in order to let overlaid points appear darker; thus, the shade of the points reflects their density in each region of the scatterplot. We updated the text and the legend of Fig 2B (lines 204-206) accordingly. For a complete picture, we also added 2D histogram plots of both the 207,326 comparisons and the 2,022 pairs as supplementary **figures S2A and S2C**.

Minor comments / typos

- l.154 the three tools MM-align, RNA-align and TM-align should have references.

We added references for these tools on lines 163-164 (as a consequence, reference numbering changed throughout the manuscript). The tools are also still referenced in the Methods, as in the previous version.

- l.158 "out of over 444000 comparisons" -> one has to check all the material and methods to understand why it is not (977*977)/2-977/2 = 476776 comparisons. A short sentence with a reference to the "Structural alignment" part in the material and methods would help here.

We added a sentence as suggested to clarify this point in the results section (lines 167-169): "In principle, we should perform 476,776 (= 977 x 976 / 2) comparisons; however, we refrained from comparing pairs of interfaces that belong to the same PDB entry and have one chain in common, as these cannot be structural interologs (see Methods). Out of over 444,000 remaining possible comparisons,…"

- l.159 MMalign succeeds in aligning 207000 pairs -> why did it not succeed for the others? What are the criteria for a successful alignment? Is it that MMalign could not align at all, or that the TM-score is < 0.5?

For a pair of protein-RNA interfaces, MM-align returns a structural alignment that does not always display correspondence between both protein chains and between both RNA chains (i.e. sometimes, the proteins are aligned, but MM-align is unable to find an alignment for the RNA chains). This is a sign of very different interfaces, and therefore is our first filter, eliminating more than half of the compared interface pairs. Analysis of the interface TM-score returned by MM-align only makes sense in the remaining ~207,000 comparisons, leading to our second filter (interface TM-score > 0.5) as displayed in Fig 2A. We modified the text to clarify this point (on lines 170-173).

- l.179 and fig.2: if I understand correctly: out of the 977 interfaces, 765 are kept and form 2022 pairs of interologs. This could be added to fig.2A to make things more clear for the reader.

Indeed, this is exactly the case. We added “2,022 pairs from 765 interfaces” in the bottom-most box in **Fig 2A** and we clarified the text on line 196: “Notably, 765 out of the 977 initial representative interfaces (78%) form the final 2,022 pairs and have at least one identified structural interolog in this process.”

- Fig S1E, S2, S3, S4 have no label for ordinate, the font and font size are not consistent intra-figures.

We made sure **all figures and supplementary figures** (including newly added ones) have appropriate x-axis and y-axis labels, and consistent fonts and font sizes.

- figS4 is poorly presented.

We simplified and reorganized Fig S4 (now **Fig S8**) to make the presentation clearer and more self-consistent.

- The authors also made available a website. I browsed it, and it is easy to use. One additional option could be, if possible, to enable the user to search for a specific PDB identifier from the home page. If one wants to see if a specific PDB structure has interologs I would rather type in the PDB code in a search bar to check if it belongs to any group. From what I understand it is currently only possible to search inside of a group.

We thank the reviewer for browsing the website and pointing out its user-friendliness. We added an option on the home page to search with a PDB code. This option highlights only groups of interologs containing interfaces from this PDB structure. From there on, these groups can be clicked to access more detailed information. The search by PDB identifier is also available on the “All interfaces” page (retrieving all interfaces belonging to this PDB structure) and on the “All interologs” page (retrieving all pairs of interologs where one interface belongs to this PDB structure).

We complemented the text accordingly on lines 521-522: “On all three pages accessible from the navigation menu of the web interface (home page, interolog group page, and interface list page), users can search for specific keywords within PDB, ECOD and Rfam descriptions of the macromolecular components”

- l. 538 I understand why clustering at 100% sequence identity for proteins, but why at 99% for RNAs?

We followed the cd-hit-est clustering threshold initially used for clustering RNA molecules in protein-RNA interfaces in ref 12 (Zheng et al, doi: 10.1371/journal.pcbi.1005120). This precision was added in the Methods on line 625: (the RNA redundancy threshold was taken from [12]). After checking, using a threshold of 100% would change only very slightly the number of RNA representative sequences included in the clustering process (13 additional representatives out of 308 total).

- l. 567: why did FREESASA fail? Is it technical? 141 interfaces is a lot.

After verifying the freesasa calculations, we were able to fix the technical issue and compute relative accessible surface area of the complex for protein chains in all 765 interfaces. Consequently, we were able to extend the analysis of contact conservation depending on core/rim assignment to all 2022 pairs of interologs.

Most importantly, **the overall conclusions are unaffected by this update**. In practice, we removed the statement about freesasa failures previously included at the end of the "Interface analysis" section; we slightly corrected the values indicated on lines 391-393 (from 86% to 85%, from 73-75% to 72-74%, p-value from <4e-46 to <4e-56); and we corrected Fig S5A (now **Fig S9A**) and its legend. We also updated the RNAprotDB web interface to include the previously missing core/rim assignment for the 141 interfaces in question.

Reviewer #3: The main objective of the study summarized in the manuscript is to explore and characterize the evolution of protein-RNA interface. To this aim the authors generated a data base of interface-interface pairs (defined as interlogs) which provide a very good resource for the community.

Overall, based on a comprehensive analysis of the interlogs (divided to groups according to the degree of conservation, the authors conclude that in general protein-RNA interfaces are more conserved than protein-protein interfaces and that the most conserved contacts are the apolar contacts while hydrogen bond are the least conserved. While this conclusion is consistent with many previous studies it provides additional insights, which are supported by a relative large dataset. Finally, the authors built a user friendly and informative web server, which can be of great contribution to the protein-RNA community.

We thank Reviewer #3 for their overall very positive assessment of our work. We provide point-by-point replies to the detailed remarks below (including ribosomal vs. non-ribosomal analysis and analysis of specific interolog families).

Below are my detailed remarks: (we numbered the points for an easier response)

1- As stated, the main aim of this work is to study protein-RNA interface evolution. Given that the majority of the interfaces are from the ribosome the authors correctly divide the results to ribosomal and non-ribosomal interfaces. However, given the importance of this grouping for the interpretation of the results, it is critical that the results of each group are discussed

separately and preferably presented in a main figure. Moreover, I do not find that the violin plot showing the results summarized for all sub groups (based on conservation level) are of any value. What can one learn from looking at % of conserved contact when the level of conservation range between 10 to 90% ?

To address both points raised by the reviewer in this remark, we replaced former Fig 3C and Fig 3D by the contact conservation violin plots split between ribosomal and non-ribosomal interfaces and by sequence identity groups (former Fig S3C and S3D). We split former Fig 3 into Fig 3A-B explaining how contact conservation is computed, and current **Fig 4** showing the ribosomal/non-ribosomal distributions of contact conservation. We moved the full distributions to supplementary figures (to show the wide variety of situations). We also moved stacking conservation distributions to supplementary figures, since there are too few points to split them meaningfully. We updated and reorganized the manuscript text to accommodate the deeper focus given to ribosomal/non-ribosomal interface tendencies. In particular, we added explicit description and discussion of the separate distributions of contact conservation for ribosomal vs. non-ribosomal interolog pairs in the results section of the main text (lines 280-284 and 304-313).

2- The conclusions of this study are based on the analysis of the two major interface groups (ribosomal and non-ribosomal). Given that these results are suggested/expected to provide key features for modeling it is critical to show how/whether the relative conservation differs between interfaces coming from different RNA binding protein families (based on Rfam). As for example one would expect that the interfaces from RRM will differ from those that bind double strand RNA. Previous studies, as for example PMID: 25932908, have shown that the interfaces of the RNA bound to the different protein families have unique properties. It is thus critical that the author repeat their analyses upon groupding the interfaces based on the main RBP domain involved in the interface. As is, the authors should be much more cautious in the interpretations of the results and tone down their conclusions regarding the contribution of their study and its potential to be implemented for molecular modeling and docking. I suspect that the current analysis is not sufficient to provide useful scoring functions for protein-RNA modeling.

We thank the reviewer for this very interesting remark. As previously described, we grouped interologs by connecting them through structural similarity relationships, and we found that the obtained 141 groups are self-consistent in terms of ECOD/Rfam family annotation. One major limitation of a family-specific analysis is that most of these interolog groups are very small (e.g. 54 groups contain 1, 2 or 3 interolog pairs). Among the larger groups, we can identify well-known families (including several examined in PMID: 25932908) such as RRM, RNAse H-like (including Ago), RNA helicases, viral RNA polymerases, and some large ribosomal groups such as L30e-like, L10e with LSU, or SRP. In the main text, we added discussion about some of these large groups (lines 328-360). We added distributions of contact conservation for four of the largest groups (RRM, RNase H-like, L10e with LSU and RNA helicases) in supplementary **Fig S7**. Importantly, this remark brought us to enrich the Jupyter notebook associated with the manuscript in order to enable analysis of contact conservation in any specific group of interologs from our dataset. This complements the analysis provided by our RNAprotDB web interface where interolog groups can be dynamically explored.

3- The comparison between “non-conserved contacts” and “switching out of the interface” seems confusing to me, as to my understanding there is a strong dependency between the two

and both are strongly dependent on the interface and interlog definitions, which were delineated by the authors.

In the study, we analyze contacts that are not conserved between interologs (i.e. contacting aa/nt pair in one interolog, but the aligned aa/nt pair does not interact). There are several possible scenarios for this non-conservation, and we tried to disentangle them in view of future prediction perspectives (e.g. trying to transfer contacts from an interface structure to a homolog). The "switching out" scenario is the most extreme, when an aa or nt not only loses the contact we are considering but also all other contacts (it does not make any contacts in the interolog). As the interface is defined by contacts in our study, this translates into the residue no longer being involved in the interface. We clarified this point in the abstract (line 35) and on lines 408-411. We also rephrased our analysis to remove the (possibly misleading) mentions of a "causal" link between switching out and non-conservation, e.g. on lines 418-419: "we found that switching out was involved in a large proportion of non-conserved atomic contacts, 47% on average".

As we report, "switching out" affects a small fraction of interface residues but a large fraction of the non-conserved contacts. This means that in a prediction perspective, it might be interesting to identify those residues before predicting the conservation of each interface contact. As this point is one of the reasons why we focus on "switching out", we added a paragraph to explain it (lines 425-429):
"Because switching out affects a small fraction of interface residues but a large fraction of the non-conserved contacts, identifying switching out residues could be interesting in a prediction perspective, before considering the conservation of each interface contact. Switching out residues display characteristic features that would be useful in this regard; for instance, 26% of (unweighted) amino acids from the rim interface region switch out, as opposed to 17% from the core region."

4- I find the following sentences either misleading or extremely over estimated and should be rephrased/toned down

4-1. "we focused on the analysis of protein-RNA interface evolution, aiming to unravel relevant keys for structural modeling and prediction of protein-RNA complexes and for interpretation of multiple sequence alignments"
This sentence does not adhere to the results presented in this study.

We rephrased this sentence to better reflect the results presented in our study (line 129): "In the present study, we focused on the analysis of protein-RNA interface evolution, aiming to unravel the sequence and structural properties connected with interface conservation and plasticity." We now mention the possible applications of our work only in the discussion.

4-2. "Numerous studies show that hydrogen bonds play a crucial role in protein-RNA interactions by contributing to the specificity and stability of the complexes and the function of RNA-protein complexes [45, 46]. "

The references cited are not suitable. There are many other key references that are clearly more relevant (several that are cited in the manuscript) and should replace the ones cited.

Reference 45 (now ref 16) describes hydrogen-bonding potentials for protein-RNA interface design and docking, and was moved to the introduction in the sentence about statistical potentials (line 88): "Some of the most common scoring approaches rely on statistical potentials, deriving pairwise residue-ribonucleotide propensities [13-15] or hydrogen-bonding geometries [16] from known structures of protein-RNA interfaces."

We rephrased the sentence mentioned by the reviewer (line 443) "Numerous studies show that hydrogen bonds play a crucial role in protein-RNA interactions by contributing to the specificity, stability and function of the complexes [40, 42, 53, 54]." We left in ref 46 (now ref 53) which is a detailed study of hydrogen bonding in 300 protein-RNA interface structures. We added references to Corley et al (former ref 40, now ref 42), Treger & Westhof (former ref 38, now ref 40) and a new reference to Barik, … Bahadur J Biomol Struct Dynamics 2015 (new ref 54 = PMID 25562181, describing the contribution of H-bonds to protein-RNA interfaces).

4-3. "…as the ability to form H-bonds through the amino acid backbone does not vary with evolutionary mutations."

This sentence is inaccurate and contradicts some of the message in the paper regarding the evolution of interfaces and changes in geometry that can surely affect the h-bonding via the protein backbone atoms.

We agree with the reviewer that this sentence was misleading and we rephrased it to explain the reason for focusing on H-bonds involving amino acid side chains (lines 447-448): "In this analysis, we focused only on H-bonds involving the side chains of amino acids; this simplifies the interpretation of possible compensation scenarios involving evolutionary mutations affecting the amino acid nature."

4-4 "…and we evidenced increased contact conservation for evolutionarily more conserved interface regions. This latter feature is especially interesting since it does not require prior experimental knowledge of a protein-RNA interface structure."

The observation that increased contact conservation for evolutionarily more conserved interface regions seems very trivial. Do the authors mean that there is increased contact conservation for evolutionarily more conserved surface regions? this is a very important message and critical for predicting protein-RNA interfaces.

There are actually two trends linked to evolutionary conservation, as derived from a protein multiple sequence alignment.

(1) It is well known that binding regions are more conserved than the non-interacting protein surface; hence, evolutionary conservation can be used as a feature to predict protein-RNA interfaces. We added this information explicitly in the introduction, together with a new reference (lines 102-104): "Alongside the propensity of amino acids (in particular positively charged residues) for binding RNA, evolutionary conservation derived from protein MSAs is one of the major features enabling the prediction of RNA-binding residues [27]."

(2) In our study, contact conservation is only defined for interface regions, as by definition, any residue making a contact belongs to the interface. Therefore, we were wondering if within the (generally well-conserved) interface regions, some amino acids with even higher evolutionary conservation specifically conserved their contacts in homologous interfaces more often than the rest of the interface. This is indeed the case, and we show the result of this analysis in the manuscript (Results, lines 396-404; **Fig S9B**).
One goal of our analysis is to define interesting features that can be used in prediction; e.g. when doing homology modeling, to know which contacts can be transferred from the known protein-RNA interface to the target homologous complex. These features (properties of the target complex) ideally should not depend on any prior knowledge about the target interface. In this respect, evolutionary conservation is very interesting, because it can be computed without knowing the target interface structure (e.g. mapped onto an AlphaFold model of the protein in the target complex).
We clarified the corresponding lines 561-567 in the discussion (pointed out by the reviewer): "We evidenced increased contact conservation for evolutionarily more conserved interface regions, meaning that the most conserved interface amino acids also have the most contact conservation. This latter feature is especially interesting in a prediction context, e.g. when wondering which contacts can be transferred from a known protein-RNA interface to a target homologous complex, since computing evolutionary conservation and mapping it onto the protein surface does not require prior experimental knowledge about the target protein-RNA interface structure."

4-5. The author claim that "we offer on protein-RNA interface evolution for a diverse range of protein and RNA families open new possibilities for tailoring scoring metrics to further improve protein-RNA specificity predictions" however, the paper they cite actually show that a general scoring function is not sufficient and family specific functions are required. This point is critical and should be emphasized.

We clarified this discussion point and complemented it, following the analysis carried out in response to remark #2 from this reviewer (lines 571-577):
"Additionally, our analysis and the resources we provide cover a diverse range of protein and RNA families. For the largest analyzed interolog groups, our carefully crafted dataset and our insights on protein-RNA interface evolution open new possibilities for tailoring family-specific scoring metrics to further improve protein-RNA specificity predictions. As an illustration of such specificity studies, a recent study used curated sequence and structure data to gain insights into the recognition code for RRM-RNA interactions and derive a specificity prediction score for this family [49]."
